# High level of intrinsic phenotypic antimicrobial resistance in enterobacteria from terrestrial wildlife in Gabonese national parks

**DOI:** 10.1371/journal.pone.0257994

**Published:** 2021-10-12

**Authors:** Pierre Philippe Mbehang Nguema, Richard Onanga, Guy Roger Ndong Atome, Jean Jules Tewa, Arsène Mabika Mabika, Jean Ulrich Muandze Nzambe, Jean Constant Obague Mbeang, Paul Yannick Bitome Essono, François Bretagnolle, Sylvain Godreuil

**Affiliations:** 1 Departement Ecologie Animal, Institut de Recherche en Ecologie Tropicale (IRET), Libreville, Gabon; 2 Centre Interdisciplinaire de Recherches Médicales de Franceville (CIRMF), Franceville, Gabon; 3 UMR CNRS/uB 6282 Biogéosciences, Université de Bourgogne-Franche-Comté, Dijon, France; 4 Department de Chemie, Faculté des Sciences, Université des Sciences et Techniques de Masuku (USTM), Franceville, Gabon; 5 Departement de Mathematiques et Informatique, Faculté des Sciences, Université de Douala, Douala, Cameroun; 6 Institut de Recherche en Technologie (IRT), Libreville, Gabon; 7 Centre Hospitalier Universitaire de Montpellier, Laboratoire de Bactériologie, Université de Montpellier, Montpellier, France; Nitte University, INDIA

## Abstract

Data on the prevalence of antibiotic resistance in *Enterobacteriaceae* in African wildlife are still relatively limited. The aim of this study was to estimate the prevalence of phenotypic intrinsic and acquired antimicrobial resistance of enterobacteria from several species of terrestrial wild mammals in national parks of Gabon. Colony culture and isolation were done using MacConkey agar. Isolates were identified using the VITEK 2 and MALDI-TOF methods. Antibiotic susceptibility was analysed and interpreted according to the European Committee on Antimicrobial Susceptibility Testing guidelines. The preliminary test for ESBL-producing *Enterobacteriaceae* was performed by replicating enterobacterial colonies on MacConkey agar supplemented with 2 mg/L cefotaxime (MCA+CTX). Extended-spectrum beta-lactamase (ESBL) production was confirmed with the double-disc synergy test (DDST). The inhibition zone diameters were read with SirScan. Among the 130 bacterial colonies isolated from 125 fecal samples, 90 enterobacterial isolates were identified. *Escherichia coli* (61%) was the most prevalent, followed by *Enterobacter cloacae* (8%), *Proteus mirabilis* (8%), *Klebsiella variicola* (7%), *Klebsiella aerogenes* (7%), *Klebsiella oxytoca* (4%), *Citrobacter freundii* (3%), *Klebsiella pneumoniae* (1%) and *Serratia marcescens* (1%). Acquired resistance was carried by *E*. *coli* (11% of all *E*. *coli* isolates) and *E*. *cloacae* (3% of all *E*. *cloacae*) isolates, while intrinsic resistance was detected in all the other resistant isolates (n = 31); *K*. *variicola*, *K*. *oxytoca*, *K*. *pneumoniae*, *E*. *cloacae*, *K*. *aerogenes*, *S*. *marcescens* and *P*. *mirabilis*). Our data show that most strains isolated in protected areas in Gabon are wild type isolates and carry intrinsic resistance rather than acquired resistance.

## Introduction

The emergence of antibiotic resistant bacteria (ARB) in the *Enterobacteriaceae* family is a major issue worldwide that affects the dynamics of microbial populations and leads to human public health problems [1]. Antibiotic resistance is genetically encoded, but can be intrinsic or acquired. Intrinsic resistance describes the innate capacity of a bacterial species to resist to a specific drug. Conversely, acquired resistance is found only in some isolates of a bacterial species and results from horizontal gene transfer, or more rarely, from selection of a mutation. For more than 50 years, ARB studies have focused on pathogenic bacteria isolated from hospitals and more recently from rural environments. Indeed, the massive use of antibiotics in farming has strongly increased ARB prevalence in agricultural zones [2, 3]. Moreover, many studies have documented the prevalence of resistance in wild animals. This phenomenon has been largely interpreted as the result of contacts with contaminated anthropogenic sources [4]. However, the discovery of resistant *Enterobacteriaceae* strains carried by wild animals or in the environment, outside areas frequented by humans and for which human contamination seems unlikely, suggests that resistance might be present in environmental reservoirs and has an adaptive significance that predates the antibiotic era [5–7]. The existence of environmental reservoirs of resistance, with the possibility of transferring resistance from the wildlife compartment to humans and domestic animals, raises the problem of the wildlife role in the dynamics of antibiotic resistance emergence. Multi-resistant bacteria (i.e. the bacteria which are non-susceptible to at least one antimicrobial agent in three or more antimicrobial classes [8]) in wildlife are good markers for assessing the transfer dynamics between humans and wildlife because it is thought that these isolates are selected in anthropogenic environments and then transferred to wildlife [9, 10]. However, studies on the prevalence of antibiotic resistance in *Enterobacteriaceae* in African wildlife are still relatively limited. Most have focused on anthropized (urban or agricultural) areas or on emblematic species (e.g. apes). Recent studies have examined resistance in *Enterobacteriaceae* from apes with conflicting results. For example, transfer of antibiotic-resistant *Enterobacteriaceae* has been demonstrated between humans and chimpanzees in Uganda [11]. Conversely, in the Taï forest of Ivory Coast, very few resistant *Enterobacteriaceae* strains have been detected in fecal samples of chimpanzees, probably due to the low level of contact with humans and the important health precautions taken by researchers working on chimpanzee groups [12]. In Gabon, antibiotic resistance in wildlife in protected areas is low (e.g. ampicillin: 3.4% of *Escherichia coli* isolates from gorillas, 8.3% from other wildlife; streptomycin: 2.5% of *E*. *coli* isolates from gorillas, 2.1% from other wildlife; tetracycline: 2.5% of *E*. *coli* isolates from gorilla, 4.2% from other wildlife) [13], but high in fruit bats in unprotected areas (e.g. ampicillin: 100%; streptomycin: 100%; tetracycline: 83.33% of *E*. *coli* isolates) [14]. Therefore, the aim of this study was to estimate the prevalence of phenotypic intrinsic and acquired enterobacterial antimicrobial resistance in different wild terrestrial mammals in several national parks of Gabon.

## Materials and methods

The research license for this study was obtained from the Scientific Commission for Research Authorization of the National Center for Scientific and Technological Research (CENAREST) (permit no. AR0019/15/MESRS/CENAREST/CG/CST/CSAR, dated July 9, 2015). Authorization to access national parks was granted by the National Parks Agency (ANPN) (permit No. AE15014/PR/ANPN/SE/CS/AEPN, 9 July 2015).

Fecal samples were collected in Moukalaba Doudou National Park (MDNP), Loango National Park (LONP), Lope National Park (LPNP) and Lékédi Private Park (LPP) in August 2015 (LPNP), May 2016 (LPP), July 2016 (MDNP), and July 2016 (LONP). Wildlife feces were collected non-invasively by following wild mammals in the forest and collecting the excrements they left behind. Feces were collected either after immediate defecation or three hours after defecation, determined by observation of their color, temperature and consistency. To avoid environmental contamination, in the forest, only feces that were not covered by dust and were preferably deposited on leaves on the ground were collected. Feces from the following animals were collected: *Gorilla gorilla gorilla*, *Mandrillus sphynx*, *Cercocebus torcatus*, *Cercopithecus nictitans*, *Colobus satanus*, *Cephalophus sp*., *Genetta genetta*, *Kobus ellipsiprymnus*, *Loxodonta cyclotis*, *Syncerus caffer*, and *Potamocherus porcus* (Table 1). Each fecal sample was placed in a small sterile plastic bag using gloves and wooden tweezers (a new pair of tweezers for each sample), and then stored in a large bag in a dark place. In the laboratory of the camp site, each sample was cut with sterile tweezers and a small amount of feces was collected from the middle and streaked on a 60 mm MacConkey agar (MCA; bioMérieux, France) plate and incubated at 37°C for 24h, according to a previously established protocol [15, 16]. After incubation, each colony morphology was recorded (structure and color), and then the colony was picked, transferred to phosphate buffered saline (PBS) supplemented with 30% glycerol for storage in ambient conditions during the feces collection period (7 days).

**Table 1:** The different antimicrobial resistance phenotypes in enterobacterial isolates from fecal samples collected in national parks of Gabon.

Mammal (n)	*Bacterium*	Phenotype											Type of resistance
*Colobus satanus (1)*	*E*. *coli*	LEV											acquired
*Gorilla gorilla gorilla (1)*	*E*. *coli*	AMX	TIC										acquired
*Gorilla gorilla gorilla (1)*	*E*. *coli*	NAL	CHL	TET									acquired
*Mandrillus sphynx (1)*	*E*. *coli*	AMX	TIC	CHL	SXT								acquired
*Mandrillus sphynx (1)*	*E*. *cloacae*	AMX	AMC	ATM	TIC	TIM	PRL	TZP	CFL	FOX	CTX	CAZ	acquired
*Gorilla gorilla gorilla (2)*	*P*. *mirabilis*	TET											intrinsic
*Mandrillus sphynx (1)*	*P*. *mirabilis*	TET											intrinsic
*Syncerus caffer (1)*	*P*. *mirabilis*	TET											intrinsic
*Gorilla gorilla gorilla (2)*	*K*. *oxytoca*	AMX	TIC	PRL									intrinsic
*Syncerus caffer (1)*	*K*. *oxytoca*	AMX	TIC	PRL									intrinsic
*Potamochoerus porcus (1)*	*K*. *oxytoca*	AMX	TIC	PRL									intrinsic
*Gorilla gorilla gorilla (1)*	*K*. *pneumoniae*	AMX	TIC	PRL									intrinsic
*Gorilla gorilla gorilla (2)*	*K*. *variicola*	AMX	TIC	PRL									intrinsic
*Mandrillus sphynx (2)*	*K*. *variicola*	AMX	TIC	PRL									intrinsic
*Potamochoerus porcus (1)*	*K*. *variicola*	AMX	TIC	PRL									intrinsic
*Loxodonta cyclotis (1)*	*K*. *variicola*	AMX	TIC	PRL									intrinsic
*Syncerus caffer (2)*	*K*. *aerogenes*	AMX	AMC	CFL	FOX								intrinsic
*Gorilla gorilla gorilla (4)*	*K*. *aerogenes*	AMX	AMC	CFL	FOX								intrinsic
*Gorilla gorilla gorilla (4)*	*E*. *cloacae*	AMX	AMC	CFL	FOX								intrinsic
*Cercopithecus nictitans (1)*	*E*. *cloacae*	AMX	AMC	CFL	FOX								intrinsic
*Mandrillus sphynx (1)*	*E*. *cloacae*	AMX	AMC	CFL	FOX								intrinsic
*Mandrillus sphynx (3)*	*C*. *freundii*	AMX	AMC	CFL	FOX								intrinsic
*Mandrillus sphynx (1)*	*S*. *marcescens*	AMX	AMC	TIC	CFL								intrinsic

AMX, amoxicillin; AMC, amoxicillin+clavulanic acid; ATM, aztreonam; CAZ, ceftazidime; CFL, cephalexin; CHL, chloramphenicol; CTX, cefotaxime; FOX, cefoxitin; LEV, levofloxacin; NAL, nalidixic acid; PIR, piperacillin; SXT, trimethoprim/sulfamethoxazole; TEM, temocillin; TIC, ticarcillin; TIM, ticarcillin+clavulanic acid; TZP, piperacillin+tazobactam; MDNP, Moukalaba Doudou National Park; LONP, Loango National Park; LPNP, Lopé National Park; LPP, Lékédi Private Park.

In the bacteriology laboratory of the Interdisciplinary Medical Research Center of Franceville (CIRMF) (Franceville, Gabon), colonies were streaked on the same medium. The preliminary test for ESBL-producing *Enterobacteriaceae* was performed by replicating enterobacterial colonies on MCA supplemented with 2 mg/L cefotaxime (CTX) (MCA/CTX). Compared with not supplemented MCA, the MCA/CTX combination significantly increases the detection of resistance to beta-lactam antibiotics [17], and the selection of intrinsic and acquired beta-lactam resistant bacteria, such as those producing extended-spectrum beta-lactamases (ESBL). The bacterial colonies were identified with the VITEK 2 system (bioMérieux) and MALDI-TOF (Bruker Daltonics, Bremen, Germany). Antibiotic susceptibility testing was performed with the agar disc diffusion method. The following antibiotics, often used to treat human bacterial infections in local clinics, were tested: amoxicillin (25 μg), amoxicillin–clavulanic acid (20 and 10 μg, respectively), aztreonam (30 μg), cefepime (30 μg), cefotaxime (30 μg), cefoxitin (30μg), ceftazidime (30 μg), cephalexin (30 μg), chloramphenicol (30 μg), ertapenem (10 μg), fosfomycin (200 μg), gentamicin (10 μg), imipenem (10 μg), levofloxacin (5 μg), nalidixic acid (30 IU), netilmicin (10 μg), ofloxacin (5 μg), piperacillin–tazobactam (30 and 6 μg, respectively), piperacillin (30 μg), temocillin (30 μg), tetracycline (30 μg), ticarcillin–clavulanic acid (75 and 10 μg, respectively), ticarcillin (75μg), tobramycin (10μg), and trimethoprim–sulfonamide (1.25 and 23.75 μg, respectively). ESBL production was tested with the double-disc synergy test (DDST). The set of beta-lactam antibiotics was tested simultaneously on the same antibiogram to determine the acquired or intrinsic phenotype [18–21]. The inhibition zone diameters were read and interpreted with SIRscan (i2a, France) following the recommendations of the European Committee on Guidelines for Antimicrobial Susceptibility Testing (EUCAST) (version 7.1).

## Results

In total 90 enterobacterial isolates were identified among the 130 colonies from the 125 fecal samples collected in national parks of Gabon. *E*. *coli* (61%, 55/90) was the most prevalent, followed by *Enterobacter cloacae* (8%, 7/90), *Proteus mirabilis* (8%, 7/90), *Klebsiella variicola* (7%, 6/90), *Klebsiella aerogenes* (7%, 6/90), *Klebsiella oxytoca* (4%, 4/90), *Citrobacter freundii* (3%, 3/90), *Klebsiella pneumoniae* (1%, 1/90) and *Serratia marcescens* (1%, 1/90).

Only one colony of enterobacteria was observed on MCA/CTX. The DDST did not detect any ESBL-producing isolate among the 90 isolates. Moreover, 60% (52/90) of these enterobacterial isolates were susceptible to all the antibiotics tested, particularly *E*. *coli* (56%, 51/90) and *P*. *mirabilis* (3%, 3/90). Four *E*. *coli* isolates (11%, 4/36) from different monkey and ape species showed acquired resistance to amoxicillin, ticarcillin, chloramphenicol, nalidixic acid, tetracycline, trimethoprim–sulfamethoxazole and levofloxacin (Table 1). One *E*. *cloacae* isolate (the colony that grew up on MCA/CTX) (3%,1/36) from mandrills was resistant to amoxicillin-clavulanic acid, aztreonam, piperacillin, piperacillin-tazobactam, cefalexin, cefoxitin, cefotaxime, ceftazidime and cefepime. But DDST on this isolate (*E*. *cloacae*) did not reveal a synergy image that suggested ESBL production. All the other resistant isolates carried intrinsic resistance. Specifically, *K*. *variicola* (17%, 6/36), *K*. *oxytoca* (11%, 4/36), *K*. *pneumoniae* (3%, 1/36) isolates were resistant to amoxicillin, ticarcillin and piperacillin. *E*. *cloacae* (17%, 6/36), *K*. *aerogenes* (17%, 6/36), *C*. *freundii* (8%, 3/36) and *S*. *marcescens* (3%, 1/36) were resistant to amoxicillin, amoxicillin–clavulanic acid, cefalexin and cefoxitin. *P*. *mirabilis* (11%, 4/36) were resistant to tetracycline (Table 1). All isolates harboring intrinsic resistance were wild-type (predominant bacterial strains in the natural environment).

## Discussion

We screened antibiotic resistance in enterobacterial isolates from fecal samples of wild terrestrial mammals in Gabon natural parks [17]. The use of a culture medium supplemented with a third generation cephalosporin like MCA/CTX increases significantly the detection of beta-lactam antibiotic resistance, especially in samples from asymptomatic animals [17]. The exact proportion of resistant isolates in wildlife is unknown [22], and may be very limited or non-existent in the gastrointestinal tract of wildlife in protected areas [13]. The lack of detection of ESBL-producing enterobacteria suggests their absence in the gastrointestinal tract of wildlife in Gabon national parks. The prevalence of other resistant bacteria in this study was low. Moreover, most of the resistant bacteria harbored intrinsic resistance that could be attributed to the natural resistome circulating in the environment. The few studies carried out in protected forest areas in West and Central Africa described a similar pattern of low prevalence of resistance, mostly due to intrinsic resistance, in the same enterobacterial species identified in the present study (*E*. *coli*, *Klebsiella spp*, *C*. *freundii*, *Enterobacter spp*, *P*. *mirabilis* and *S*. *marcescens*) [12, 15, 23, 24]. These bacteria are common in fecal samples of wildlife [24, 25]. *P*. *mirabilis* has an intrinsic resistance to tetracycline and colistin [26, 27]. *K*. *pneumoniae*, *K*. *oxytoca* and *K*. *variicola* are intrinsically resistant to amoxicillin, ticarcillin, and piperacillin, *Serratia*, *Enterobacter* and *Citrobacter* show intrinsic resistance to first-generation aminopenicillins and cephalosporins [19, 28]. In our fecal samples, resistant or multi-resistant *E*. *coli* isolates were found in different monkey species, although at a low rate. *E*. *coli* is naturally susceptible to several antibiotics, particularly the beta-lactam family [19, 29], and this explains why all resistance in this species is acquired [19, 29]. Several authors have suggested that the presence of resistance and particularly of multi-resistance in wildlife is the result of transfer from humans or domestic animals via contaminated sources [4, 12, 30]. However, in the study carried out in the Lopé Park in Gabon, Benavides et al (2012) detected differences between the genetic background of resistant *E*. *coli* isolates found in gorillas and in human populations living around the park. This led to the hypothesis that the presence of multi-drug resistance in wildlife may have a non-anthropic origin [31, 32]. In the present study, we did not determine the genetic background of the collected isolates. The low rate of acquired antibiotic resistance, such as *E*.*coli* multiresistant, in wildlife could be partly attributed to the fact that wild mammals have never been treated with antibiotics [12, 13], and to the low human penetration in the parks [11, 33] that makes the transfer of resistance determinants unlikely.

The large predominance of intrinsic antibiotic resistance in enterobacterial isolates from wildlife of the national parks of Gabon suggests that in such protected areas, anthropogenic contamination is still limited, possibly due to the current environmental protection policy in Gabonese conservation zones [34].

## Supporting information

S1 Data (XLSX)
